# From biocides to biology: multispecies biofilms as a sustainable, self-regenerating, and effective antifouling strategy

**DOI:** 10.1128/aem.01609-25

**Published:** 2025-10-16

**Authors:** Raphaël Lami

**Affiliations:** 1 Sorbonne Université, Université de Perpignan Via Domitia, CNRS, UMR 8176 Laboratoire de Biodiversité et Biotechnologies Microbiennes (LBBM), Observatoire Océanologique de Banyuls-sur-Mer 56736, Banyuls-sur-Mer, France; University of Delaware, Lewes, Delaware, USA

**Keywords:** antifouling, biofilms, biofouling

## Abstract

Finding antifouling strategies that are effective and environmentally safe remains a central challenge for maritime operations and ecosystem protection. Amador et al.’s article in *Applied and Environmental Microbiology* (91:e01392-25, 2025, https://doi.org/10.1128/aem.01392-25) proposes a bioinspired, applied-microbial-ecology solution: deliberately shaping pioneer biofilm communities, so they form a physical barrier against macrofouler settlement, avoiding biocides and low-adhesion inert coatings. Though focused on the ocean, this paradigm could inform broader anti-biofilm interventions across microbiology, reframing control as ecological steering rather than chemical suppression or materials-based design.

## COMMENTARY

**Fig 1:**
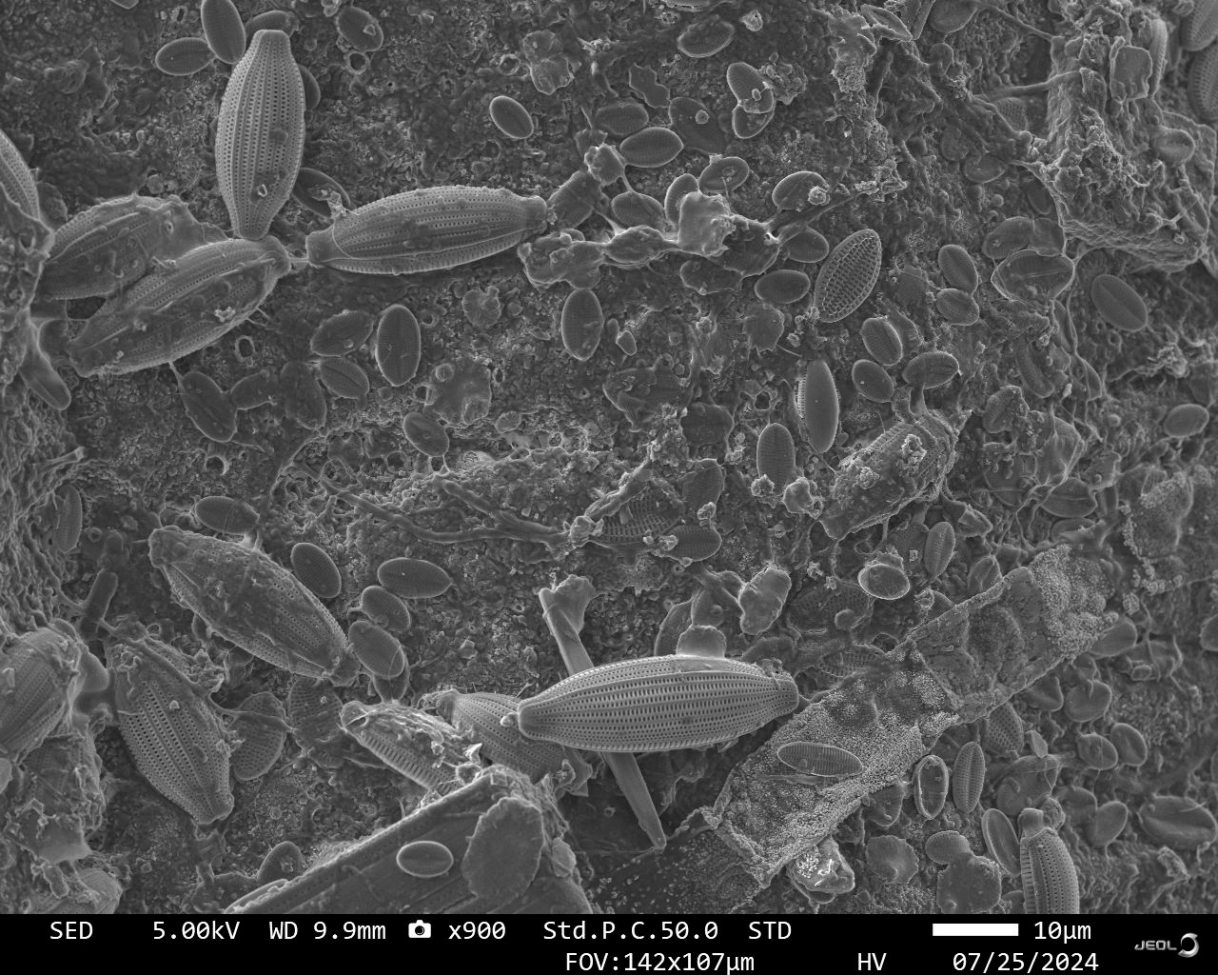
Observation of a marine microbial biofilm under electron microscopy. A variety of diatoms can be easily observed, embedded in an organic matrix. (Image courtesy of Camille Ferré, Sorbonne Université, reprinted with permission.)

Maritime operations are profoundly affected by the development of biofilms and macrofouling, the natural colonization of any immersed surface. The process is sequential: first, attachment of microorganisms (bacteria and microalgae) and construction of an extracellular polymeric substance matrix (Fig. 1); then the arrival and growth of larger organisms (e.g. barnacle larvae and macroalgal spores), culminating in an encrusting fouling layer that is particularly difficult to remove. Beyond aesthetics, the economic implications are substantial: corrosion and structural degradation, increased hydrodynamic drag and thus fuel consumption, fouling of sensors and scientific equipment, added load on offshore wind turbines, and damage to pipelines and subsea networks, to name only a few examples (1–3).

Yet despite decades of effort, no commercial solution currently reconciles operational performance with environmental safety. Manual hull cleaning is possible but not scalable. Most antifouling paints rely on biocidal compounds that raise environmental and human health concerns (even though organotins have been banned and formulations have evolved, as well as regulations). In parallel, foul-release coatings (silicones and polydimethylsiloxane [PDMS]) reduce initial adhesion and facilitate detachment under shear; they perform well for certain organism classes and at certain speeds, but their efficacy depends on the hydrodynamic regime, and some additives may also have environmental impacts.

Numerous other strategies for antifouling coating design are under active investigation but are not yet deployable at scale. These include antibiofilm but non-biocidal antifouling compounds, micro- and nanotextured biomimetic surfaces, zwitterionic polymers and hydrogels that form anti-adhesive hydration layers, and slippery liquid-infused porous surfaces, valued for their self-healing properties. Each of these approaches offers concrete advantages, but they remain challenged by the remarkable diversity of marine species, harsh offshore conditions (invasive natural biofilms, shear, high or low oceanic temperatures), and significant application and maintenance costs (4–6).

Within this context, the study by Amador et al. (7) proposes an original and elegant solution rooted in bioinspired microbial ecology. The authors suggest using the pioneer biofilm itself as a protective coating. The hypothesis is pragmatic: certain microbial communities—particularly multispecies assemblages—could form a dense, uniform, and robust film that physically prevents larval attachment and the development of deleterious macrofouling. Antifouling protection would thus arise from emergent properties of the pioneer biofilm rather than from a toxic metabolite.

The experimental workflow targets these properties directly. The authors isolate and culture numerous marine bacterial strains from vessels and algae, assess their individual biofilm-forming capacity, and then combine them in pairs and triplets to identify communities that yield higher biofilm biomass than monocultures. Competitive interactions predominate overall, although partner-dependent synergies occur. Several reproducible inducing pairs are highlighted, such as H27 (*Psychrobacter* sp.) + H18 (*Microbacterium* sp.), H20 (*Pseudoalteromonas* sp.) + H15 (*Paraglaciecola* sp.), and H13 (*Marinobacter* sp.) + H27 (*Psychrobacter* sp.). Two isolates also recur in biofilm-inducing consortia: H22 (*Maribacter* sp.) and H27 (*Psychrobacter* sp.), the latter being particularly frequent in synergistic pairs. At the multispecies level, H22 often associates with other *Psychrobacter i*solates (e.g. H25, H26, and H29) to enhance biomass. Other isolates also support biofilm cohesion and surface coverage—for example, H02 (*Alteromonas* sp.), which forms relatively confluent biofilms.

The decisive test is larval settlement. Amador et al. challenge cyprids of *Amphibalanus improvisus* on single-species biofilms and on a selected triplet. Several monocultures (e.g. *Alteromonas* H02, *Maribacter* H22, and *Psychrobacter* H25/H26) already reduce settlement compared with a polyvinyl chloride (PVC) control. Most notably, the H22 + H25 + H02 triplet drives larval settlement to nearly zero within 48 h under controlled conditions. To disentangle physical from potential chemical effects, the authors also evaluate planktonic effluents (without a surface-attached biofilm): no comparable inhibition is observed, consistent with the hypothesis of a physical barrier rather than a diffusible metabolite. Epifluorescence microscopy images corroborate the dense and uniform coverage achieved by the selected communities.

In light of the current state of the art, the proof-of-concept provided by Amador et al. is significant. It contrasts with bioinspired strategies currently pursued in many laboratories: isolating anti-biofilm compounds (notably anti-quorum-sensing agents such as macroalgal furanones), designing biomimetic textures (inspired by naturally anti-adhesive surfaces), or engineering hydrated materials (zwitterions and hydrogels) (8, 9). Whereas these approaches rely primarily on chemistry (active molecules and surface energies) and/or materials physics (mechanics and topography), Amador et al. chart a different path. The novelty is not merely conceptual; it carries practical promises: self-renewal of the film through growth, repairability after micro-damage, and adaptation to environmental fluctuations, which are properties difficult to achieve with strictly inert coatings.

Nevertheless, the road to commercial applications remains long. The properties of the engineered biofilm must still be tested against other fouling species, under high shear, microabrasion, and so on. Community stability and resistance to invasion thus become central. However, the experiments reported by the authors also show that the selected multispecies communities develop substantial biofilm biomass across 10–24°C and do so reproducibly, indicating that these natural coatings hold promise for wide-ranging use.

The work of Amador et al. will undoubtedly inspire future antifouling strategies, and not only among teams focused on the maritime domain. In particular, this line of research may impact colleagues in the medical field (10), where identifying microbial communities that form harmless, protective biofilms could prove more promising than pursuing biocidal anti-biofilm substances that will, inevitably over the medium to long term, generate resistance that is difficult to curb.
